# Correction: Zhang, T.; Wang, S. Explanation of the Influence of Sodium Chloride Solution on Volume Deformation and Permeability of Normally Consolidated Clays. *Materials* 2019, *12*, 1671

**DOI:** 10.3390/ma19214603

**Published:** 2026-10-30

**Authors:** Tongwei Zhang, Shijun Wang

**Affiliations:** 1 School of Civil Engineering and Mechanics, Lanzhou University, Tianshui Road 222, Lanzhou 730000, China; 2 Key Laboratory of Mechanics on Disaster and Environment in Western China, Lanzhou University, Tianshui Road 222, Lanzhou 730000, China; 3 Economy and Technology Research Institute, Gansu Electric Power Company, State Grid, Lanzhou 730050, China; angelofkill@126.com

The journal’s Editorial Office and Editorial Board are jointly issuing a resolution and removal of the Journal Notice linked to this article [1], as well as an update to the original publication. Following concerns raised about the integrity of the peer review, the Editorial Office has conducted a post-publication peer review of this article. This process included the recruitment of a new independent reviewer, and it was supervised by an Academic Editor to ensure full compliance with MDPI’s Editorial Process (https://www.mdpi.com/editorial_process).

As a result of this process, the Academic Editor and the authors have agreed to update the following aspects of this publication:

- Reviewer 3 was removed from the peer review record, and the new report was uploaded to the peer review record (https://www.mdpi.com/1996-1944/12/10/1671/review_report).
- The Abstract is updated to “The marine clays located in the Southeast area of China are characterized by their higher water content, higher compressibility and higher salinity. This soil is mainly composed of illite/montmorillonite interlayer minerals. Previous research has shown that the saline water significantly influences the liquid limit and other physical characteristics of the clays. As the desalination of pore water occurs as a result of freshwater or rainfall, the physical and mechanical behaviors of the soft marine clays change, and this can lead to potential hazards for infrastructure. Therefore, it is essential to understand the effects of chemical variations and to predict the long-term foundation deformations. Based on previous works, the deformation behavior of artificial soils corresponding to a mixture of kaolin and bentonite (the mass ratios of bentonite were 0%, 5%, 10% and 20%) was further discussed in an ln(1 + *e*)-log*p’* system. The permeabilities of the samples mixed with different concentrations of sodium chloride solutions were compared based on oedometer tests. The microstructures in the samples were investigated by SEM (scanning electron microscopy) tests. The declining trend of a newly defined volume compression index *C*_cv_ and swelling index *C*_sv_ with pore water salinity and *e*_0_/*e*_L_ was observed when the initial void ratios *e*_0_ of the samples were close. For the bentonite-kaolin mixtures, the permeability coefficient *k* increased, whereas the slope parameter *C*_k_ = *e*/log*k* decreased, with increasing ionic concentration. Finally, the changes in volume deformations and permeabilities induced by sodium chloride solution are discussed based on ‘suction pressure’ and initial void compression at the micro-level. This paper proves that the influences of salinity on the mechanical behavior of clays are mainly attributable to the interaction between diffused double layers, and these findings are helpful for improving the constitutive model of soft clays when taking pore water chemistry changes into consideration.”
- In Section 2.1, a new paragraph has been added at the beginning of the section as follows: The parent materials comprised commercial kaolin (Jiahe, Xuzhou, China), commercial calcium bentonite (Mufeng, Zhenjiang, China), and natural Lianyungang marine clay (LYG, Lianyungang, Jiangsu Province, China). Throughout this paper, kaolin and bentonite denote the source materials, whereas kaolinite and montmorillonite denote mineral phases. Their mineralogical composition, specific gravity (*G*_s_), specific surface area (SSA), and particle characteristics are summarized in Table 1 [2,18]. The commercial powders were tested in the air-dried state, whereas the natural LYG clay had an initial water content of 54.7–65.4%. The initial water contents of the reconstituted specimens are reported in Table 2.
- A new Table 1 summarizing the mineralogical composition and physical characteristics of the parent materials has been added. Correspondingly, an explanatory paragraph has been added as the third paragraph in Section 2.1, as follows:

Laser diffraction measurements indicated that approximately 20% of the commercial kaolin and bentonite particles were smaller than 2 μm and approximately 90% were smaller than 63 μm (Figure 1). For the LYG clay, the corresponding proportions were approximately 20% and 70%, respectively. These particle-size measurements represent equivalent aggregate sizes rather than the dimensions of individual clay platelets.

**Table 1:** Mineralogical composition, specific gravity, and specific surface area of the parent clay materials.

Material	Bulk Mineralogy (wt%)						Clay-Mineral Fraction (wt%)					*G*_s_	SSA (m^2^/g)
	Calcite	K-Feldspar	Plagio-CLase	Quartz	Other	Clay Minerals	Kaolinite	Montmor- Illonite	Illite-Smectite Mixed-Layer	Illite	Chlorite		
Commercial kaolin	—	—	—	2.9	8.6	88.5	100	—	—	—	—	2.66	46
Commercial bentonite	1.9	—	11.5	19.2	—	67.4	1.4	97.7	—	—	0.9	2.73	734
Lianyungang marine clay	1.5	4.1	15.6	23.2	10.6	45.0	13.0	—	44.0	29.0	14.0	2.70	10–25

- In Section 2.1, a new paragraph has been added at the end of the section as follows: The LYG clay was used only as a field-reference material in the particle-size and plasticity comparisons. All oedometer and SEM tests were conducted on the commercial kaolin and the bentonite–kaolin mixtures.
- In Section 2.2, paragraph 2, the sentence “As illustrated in Table 1, the mixtures were remolded with distilled water (B20%K80%Micro + 0% and KMicro + 0%) and 5% NaCl solution (B20%K80%Micro-02 and KMicro-02) at 1.2*LL* (*w*_0_ = 63%) and 1.3*LL* (*w*_0_ = 43%), similar to the samples used in the oedometer tests. Samples were consolidated until 100 kPa, and then carefully trimmed to cubes with a size of 1 cm × 1 cm × 1 cm. Then, the blocks were immersed in liquid nitrogen and coated with a layer of gold to conduct SEM tests [19–21]” is updated to “As illustrated in Table 2, mixtures were remolded with distilled water (B20%K80%Micro + 0% and KMicro + 0%) and 5% NaCl solution (B20%K80%Micro + 5% and KMicro + 5%) at 1.2*LL* (*w*_0_ = 63%) and 1.3*LL* (*w*_0_ = 43%) as samples used in oedometer tests. Samples were consolidated until 100 kPa, and then carefully trimmed to cubes with a size of 1 cm × 1 cm × 1 cm. Then, the blocks were immersed in liquid nitrogen and coated with a layer of gold to conduct SEM tests [19–21].”
- In Section 3.1, paragraph 1, the sentences “The clay mineral compositions of B, K and LYG clays obtained from XRD (X-ray Diffraction) analysis were 95%, 68% and 45% [1,17]. The clay fractions (particles smaller in size than 0.002 mm, BS1377-1990) of B, K and LYG clays based on laser-based particle-size measurements were about 20%, and the proportion of fine fractions (particles passing through a 0.063 mm test sieve) was 90% for B and K clays, and 70% for LYG clay. This indicated that the clay lamellae may flocculate and exist in the form of stacks, tactoids or quasicrystals.” are updated to “As summarized in Table 1, the bulk clay-mineral contents of K, B, and LYG clay obtained from XRD (X-ray Diffraction) analysis were 88.5, 67.4 and 45 wt%, respectively [1,17]. The clay particle fractions (particles smaller in size than 0.002 mm, BS1377-1990) of B, K and LYG clay from laser-based particle-size measurements were about 20%, and the proportion of fine fractions (particles passing a 0.063 mm test sieve) was 90% for B and K and 70% for LYG clay. This indicated that the clay lamellae may flocculate and exist in the form of stacks, tactoids or quasicrystals.”
- In Section 3.1, paragraph 2, the word “kaolinite” is updated to “kaolin”.
- In Section 3.2, paragraph 6, the word “kaolinite” is updated to “kaolin”.
- In Section 3.3, paragraph 4, the word “kaolinite” is updated to “kaolin”.
- In Section 3.4, paragraph 1, the word “kaolinite” is updated to “kaolin”.
- In Section 4, several sentences and paragraphs have been updated, as follows:
  2. For kaolin, the permeability coefficients *k* and the slopes *C*_k_ = *e*/log*k* of samples had negligible differences when the pore water salinities changed. For the bentonite-kaolin mixtures, the permeability coefficients increased whereas *C*_k_ decreased with increasing pore water salinity, and both trends became more pronounced as the bentonite fraction increased.
  3. The SEM observation shows that the fabric element of bentonite-kaolin mixtures with distilled water forms a thin platelet shape, while that with 5% NaCl forms a bulky pellet shape. On the other hand, for the soils composed only of kaolin, the arrangement and pattern of the clay particles remain relatively stable with pore water salinity.

With this update, the Academic Editors are satisfied that the Editorial Process relating to this article has been completed as per MDPI’s Editorial Process policy. The Editorial Office would like to thank the authors for their collaboration during this process.

## References

[1] Zhang T., Wang S. (2019). Explanation of the Influence of Sodium Chloride Solution on Volume Deformation and Permeability of Normally Consolidated Clays. Materials.

